# Signal Fingerprinting as a Novel Diagnostic Tool to Identify Conduction Inhomogeneity

**DOI:** 10.3389/fphys.2021.652128

**Published:** 2021-03-26

**Authors:** Ziliang Ye, Mathijs S. van Schie, Natasja M. S. de Groot

**Affiliations:** Department of Cardiology, Erasmus Medical Center, Rotterdam, Netherlands

**Keywords:** sinus rhythm, mapping, conduction, electrogram analysis, cardiac surgery

## Abstract

**Background:**

Inhomogeneous intra-atrial conduction facilitates both initiation and perpetuation of atrial fibrillation (AF) and is reflected in electrogram (EGM) morphology.

**Objective:**

The primary objective of this study is to investigate regional differences in features of different EGM types during sinus rhythm (SR) and to design a patient-specific signal fingerprint, which quantifies the severity and extensiveness of inhomogeneity in conduction.

**Methods:**

Patients (*N* = 189, 86% male; mean age 65 ± 9 years) undergoing coronary artery bypass grafting (CABG) underwent high-resolution mapping of the right atrium (RA), left atrium (LA), and pulmonary vein area (PVA) including Bachmann’s bundle (BB). EGMs during 5 s of SR were classified as single potentials (SPs), short double potentials (SDPs, interval between deflections < 15 ms), long double potentials (LDPs, deflection interval > 15 ms), or fractionated potentials (FPs, ≥3 deflections). Of all SPs, differences in relative R- and S-wave amplitude were calculated (R/S ratios). Time difference between first and last deflection was determined (fractionation duration, FD) and potentials with amplitudes < 1.0 mV were labeled as low-voltage. Conduction block (CB) was defined as a difference in local activation time (LAT) between adjacent electrodes of ≥12 ms.

**Results:**

A total of 1,763,593 EGMs (9,331 ± 3,336 per patient) were classified (Table 1).

**Conclusion:**

The signal fingerprint, consisting of quantified EGM features, including the R/S ratio of SPs, the relative frequency distribution of unipolar voltages, the proportion of low-voltage areas, the proportion of the different types of EGMs, and durations of LDP and FDP, may serve as a diagnostic tool to determine the severity and extensiveness of conduction inhomogeneity. Further studies are required to determine whether the signal fingerprint can be used to identify patients at risk for AF onset or progression.

## Introduction

Inhomogeneous intra-atrial conduction facilitates both initiation and perpetuation of atrial fibrillation (AF). An inhomogeneous pattern of conduction is reflected in electrogram (EGM) morphology and causes low-amplitude, fractionated potentials (FPs) (de Groot et al., 2003). Substrate-based ablation approaches in patients with AF therefore target low-voltages areas or fractionated EGMs (Rolf et al., 2014; Kottkamp et al., 2015; Yagishita et al., 2017). These additional ablation approaches in patients with AF have not yet resulted in beneficial long-term outcomes (Yamaguchi et al., 2016; Yagishita et al., 2017). This is, however, not surprising. Inhomogeneous conduction and hence EGM fractionation can also be physiological in nature, due to, for example, tissue discontinuities caused by anatomical structures such as capillaries. In addition, different use of filter settings can create or either mask fractionation.

At present, data on physiological variation in EGM morphology throughout the atria are scarce. Recently, van Schie et al. (2020) investigated the impact of AF episodes on the relative R- and S-wave ratio of unipolar, non-FPs during sinus rhythm (SR) and demonstrated that there was a loss of S-wave amplitude in patients with AF. This reduction of S-wave amplitude was associated with a decrease in conduction velocity. We hypothesize that construction of a signal profile containing quantified features of all types of EGMs may reflect the severity and extensiveness of inhomogeneity in conduction. Such a diagnostic tool would provide an individualized arrhythmogenic substrate profile which can also be tailored to possible gender- and age-specific features of EGMs.

The goal of this study, as a first step toward construction of a diagnostic signal fingerprint, is to investigate regional differences in features of different EGM types in relation to inhomogeneous intra-atrial conduction during SR at a high resolution in a large cohort of patients without atrial tachyarrhythmias.

## Materials and Methods

### Study Population

The study population consisted of 189 adult patients undergoing coronary artery bypass grafting (CABG) in the Erasmus Medical Center Rotterdam. This study was approved by the institutional medical ethical committee (MEC2010-054/MEC2014-393) (Lanters et al., 2015; van der Does et al., 2016). Written informed consent was obtained from all patients. Patient characteristics (e.g., age, medical history, and cardiovascular risk factors) were obtained from the patient’s medical record. Only patients without a history of arrhythmias were included in the present study.

### Mapping Procedure

**FIGURE 1:**
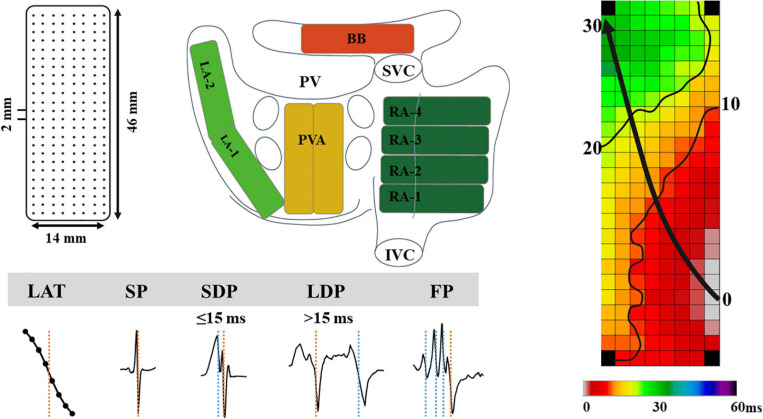
The upper left panel demonstrates a schematic presentation of the 192-unipolar electrode array and the projection of this array on a schematic posterior view of the atria. The lower left panel demonstrates typical examples of the different types of EGMs classified in this study. The right panel shows a color-coded activation map; isochrones (thin black lines) are drawn at 10 ms intervals and the black arrow indicates wavefront propagation. IVC, inferior vena cava; SVC, superior vena cava; RA, right atrium; BB, Bachmann’s bundle; LA, left atrium; PV(A), pulmonary vein (area); EGM, electrogram; SP, single potential; SDP, short double potential; LDP, long double potential; FP, fractionated potential.

As previously described, high-resolution epicardial mapping is performed before the start of extracorporeal circulation (Lanters et al., 2015; van der Does et al., 2016). Briefly, a temporary bipolar pacemaker wire was stitched to the right atrial free wall and served as a reference electrode, while a steel wire fixed to the subcutaneous tissue of the thoracic cavity was used as an indifferent electrode. Atrial epicardial mapping was performed using a 128- or 192-electrode array (electrode diameter, respectively, 0.65 or 0.45 mm, interelectrode distances 2.0 mm). Mapping was conducted by shifting the electrode array along imaginary lines with a fixed anatomic orientation, following a predefined mapping scheme, covering the entire epicardial surface of the right atrium (RA), Bachmann’s bundle (BB), pulmonary vein area (PVA), and left atrium (LA), as demonstrated in Figure 1. The RA was mapped from the cavotricuspid isthmus, shifting perpendicular to the caval veins toward the RA appendage. The PVA was mapped from the sinus transversus fold along the borders of the right and left pulmonary veins (PVA) down toward the atrioventricular groove. The left atrioventricular groove was mapped from the lower border of the left inferior pulmonary vein toward the LA appendage. BB was mapped from the tip of the LA appendage across the roof of the LA, behind the aorta toward the superior cavo-atrial junction.

Five seconds of SR was recorded at each mapping site, including a surface ECG lead, a bipolar reference EGM, and all unipolar epicardial EGMs. Data were stored on a hard disk after amplification (gain 1,000), filtering (bandwidth 0.5–400 Hz), sampling (1 kHz), and analog to digital conversion (16 bits).

### Data Analysis

Custom-made software was used to automatically measure EGM features. Missing or poor quality EGMs and premature atrial complexes or aberrant beats were excluded from analysis. The steepest negative slope of a unipolar EGM was annotated as the local activation time (LAT), providing that the amplitude of the deflection was at least two times the signal-to-noise. All annotations were manually checked with a consensus of two investigators. LATs of EGMs at each electrode were used to reconstruct color-coded activation maps (right panel of Figure 1).

As shown in the lower panel of Figure 1, EGMs were classified as single potentials (SPs, single negative deflection), short double potentials (SDPs, interval between deflections < 15 ms), long double potentials (LDPs, deflection interval ≥ 15 ms), or FPs (≥3 deflections). The time difference (ms) between the first and last deflection of FPs is defined as fractionation duration (FD). As described in our previous study, SPs were classified according their differences in relative R- and S-wave amplitudes and scaled from −1 (R-wave) to 1 (S-wave) (van Schie et al., 2020):

$$R/S=\left\{ \begin{array}{c} 1-R/S\,f\,o\,r\,R/S\leq 1\\ \frac{1}{R/S}-1\,f\,o\,r\,R/S>1 \end{array}\right.$$

Furthermore, peak-to-peak amplitudes of all potentials were measured. Low voltage potentials were, in line with prior mapping studies, defined as potentials with an amplitude < 1.0 mV (van Schie et al., 2021).

Conduction block (CB) was defined as a difference in LAT between adjacent electrodes of ≥ 12 ms (Allessie et al., 2010). Areas of simultaneous activation were excluded from analysis in order to avoid inclusion of far-field potentials.

### Statistical Analysis

Before statistical analysis, the Shapiro–Wilk test was used to determine whether the continuous variables are normally distributed. Continuous variables that are normally distributed are represented by the mean and standard deviation (SD), and differences between groups are compared by an independent sample *t*-test or least significant difference (LSD) analysis of variance. Continuous variables that are not normally distributed are represented by the median and interquartile range [IQR] or minimum to maximum. Differences between groups are compared using Wilcoxon rank sum test or Kruskal–Wallis test. Categorical variables are expressed as the number and percentages, and differences between groups are compared using chi-square test or rank sum test if appropriate. Spearman correlations were calculated to determine whether CB was associated with the EGM features. Spearman’s correlation coefficients were categorized as weak (<0.4), moderate (0.4–0.6), or strong (>0.6) correlation.

In this study, SPSS software version 20.0 and R software were used for data analysis, and *P* < 0.05 indicated significant difference. Bonferroni correction was applied to compare each of the four atrial regions with each other; a *p*-value of <0.0083 (0.05/6) was considered statistically significant (Supplementary Figure 1).

## Results

### Study Population

Baseline characteristics of the study population (*N* = 189; 86% male; mean age 65 ± 9 years) are described in Table 2. The majority of the patients had normal left ventricular function; left atrial dilatation was present in 11% of the patients.

**TABLE 1:** Overview of all signal features for each atrial region separately.

**Variables**	**RA**	**BB**	**PVA**	**LA**
Median voltage (mV)	4.68 [3.43–5.72]	5.07 [3.16–6.99]	4.73 [2.52–6.44]	5.54 [3.28–7.30]
Low-voltage (%)	5.75 [2.31–10.64]	2.20 [0.55–8.43]	4.34 [1.23–15.87]	4.47 [1.26–9.53]
R/S ratio	0.54 [0.47–0.60]	0.63 [0.55–0.71]	0.25 [0.02–0.40]	−0.02 [−0.21–0.20]
SP (%)	83.7 [75.7–89.3]	77.4 [67.3–87.3]	81.9 [69.5–88.1]	80.6 [72.0–86.5]
SDP (%)	9.8 [5.9–13.4]	11.5 [7.2–16.3]	10.3 [6.9–16.0]	11.6 [8.7–16.2]
LDP (%)	4.0 [1.9–6.8]	3.8 [0.9–9.1]	1.6 [0.3–5.5]	1.7 [0.6–4.1]
FP (%)	1.3 [0.4–3.2]	1.4 [0.2–3.6]	0.7 [0.2–2.5]	1.3 [0.4–2.5]
FD (ms)	10.0 [8.0–14.0]	11.0 [9.0–14.0]	9.0 [8.0–12.0]	9.0 [7.0–12.0]
CB (%)	2.4 [1.2–3.8]	2.3 [1.0–5.1]	1.1 [0.2–2.8]	0.7 [0.2–1.7]

**TABLE 2:** Baseline characteristics of the study population (*N* = 189).

**Variables**	**Mean+SD/*N* (%)**
Age (year)	65.44 ± 9.26
**Age**	
– < 60 years	51(26.98%)
– ≥ 60 years	138(73.02%)
Male (*N*, %)	163(86.24%)
BMI (kg/m^2^)	28.29 ± 3.87
**BMI**	
– Normal weight (18.5–25, kg/m^2^)	38(20.11%)
– Overweight (25–30, kg/m^2^)	91(48.15%)
– Obese (≥ 30, kg/m^2^)	60(31.75%)
Hypertension (*N*, %)	113(59.79%)
Dyslipidemia (*N*, %)	77(40.74%)
Diabetes mellitus (*N*, %)	62(32.80%)
Left ventricular dysfunction (*N*, %)	42(22.22%)
Left atrial dilatation > 45 mm (*N*, %)	21(11.11%)
ACEI/ARB/AT2 antagonist (*N*, %)	131(69.31%)
Statin (*N*, %)	170(89.95%)
**Antiarrhythmic drugs**	
– Class I (*N*, %)	1(0.53%)
– Class II (*N*, %)	150(79.37%)
– Class IIII (*N*, %)	3(1.59%)
– Class IV (*N*, %)	11(5.82%)
– Digoxin (*N*, %)	2(1.07%)

*Values are presented as *N* (%), mean ± standard deviation.ACEI, angiotensin-converting enzyme inhibitors; ARB, angiotensin receptor blockers; AT2, angiotensin type 2 receptor; BMI, body mass index.*

### Classification of EGM

**FIGURE 2:**
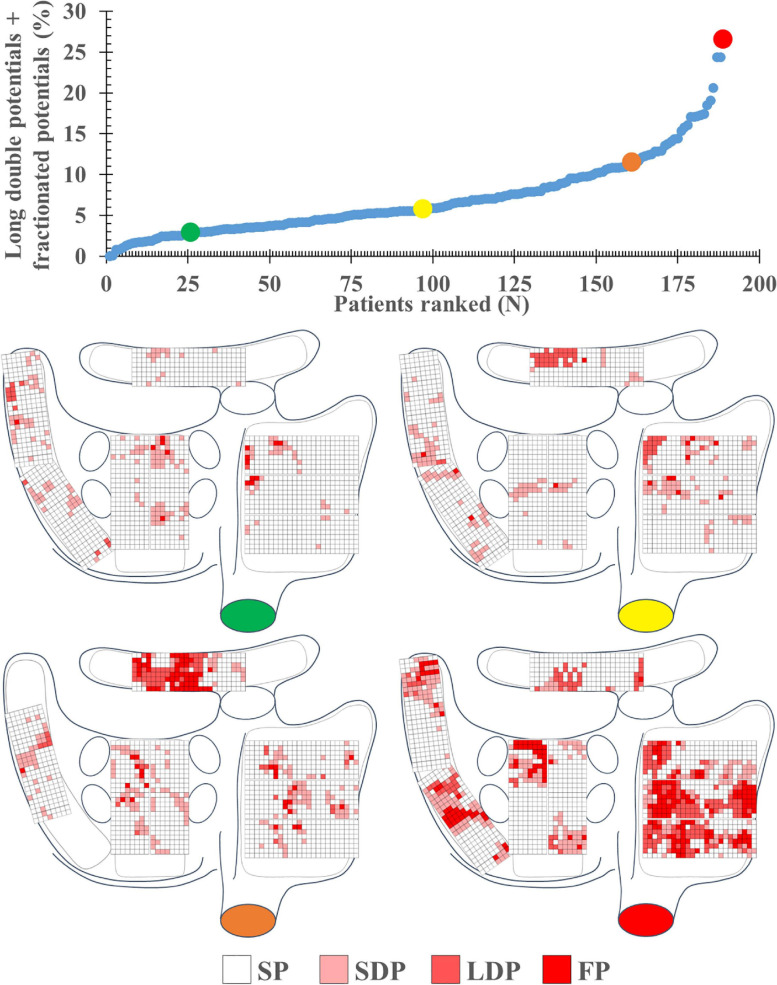
The upper left panel shows the proportion of long double and fractionated potentials for each individual patient; patients are ranked according to an increase in fractionation. The lower panel demonstrates typical examples of color-coded signal maps from four different patients, showing the proportion of the different EGM types at the various recording sites. These maps correspond to the patients marked in the graph by colored dots (green, yellow, orange, and red). FP, fractionated potential; EGM, electrogram; LDP, long double potential; SDP, short double potential; SP, single potential.

A total of 1,763,593 EGMs (9,331 ± 3,336 per patient) were analyzed; these EGMs were recorded from the RA (*N* = 841,215), BB (*N* = 196,709), LA (*N* = 343,447), and PVA (*N* = 382,222). Per region, respectively, 1.1, 4.2, 4.0, and 3.7% of the EGMs were excluded from analysis due to a poor signal-to-noise ratio. In each patient, the majority of all SR EGMs consisted of SP [81.35% (48.88–100)]. The remainder of the EGMs were mainly either SDP [10.94% (0–24.07)] or LDP [4.04% (0–20.71)]. The upper panel of Figure 2 shows the proportion of LDP+FP [5.56% (0–26.52)] plotted for each patient individually; patients are ranked according to an increase in fractionation. The lower panel of Figure 2 demonstrates typical examples of color-coded signal maps obtained from four different patients; these signal maps show the proportion of LDP and FP at every recording site. As can be seen, LDP and FP predominantly occur at the RA and BB.

### Features of EGM

**FIGURE 3:**
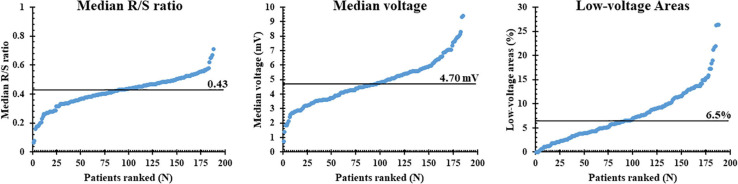
Median R/S ratios, voltage, and proportion of low-voltage areas plotted for each individual patient; patients are ranked according to increasing values of these parameters. The median value of each parameter is indicated by the horizontal line.

The left panel of Figure 3 shows the median R/S ratio of all SP per patient (*N* = 7,452 ± 2,565); the median R/S ratio was 0.43 and ranged from 0.06 to 0.71. Most EGMs (97%) had an R-wave preceding the negative deflection; only 49,896 SPs consisted of solely R- or S-waves (R-wave: 0.26%, S-wave: 3.29%). The middle and right panels of Figure 3 show, respectively, median voltage of all atrial EGMs and the prevalence of low-voltage EGMs (<1 mV) for each patient individually. Median atrial voltage was 4.7 mV and ranged from 0.7 to 9.4 mV. Low-voltage EGMs were found in the majority of the patients (98%) and accounted for 6.5% (0–26.4) of the EGMs.

### Regional Differences in EGM Features

At all atrial regions, most EGMs consisted of SPs; the proportion of SPs was significantly lower at BB and LA compared to the RA [RA: 83.7% (48.2–100) vs. BB: 77.4% (35.2–100) and LA: 80.6% (33.9–99.3); *P* ≤ 0.001 and *P* = 0.004]. The proportion of SDPs at both BB and LA was higher compared to the RA [RA: 9.8% (0–26.8) vs. BB: 11.5% (0–36.2) and LA: 11.6% (0.7–44.3); *P* = 0.039 and *P* = 0.001], whereas the proportion of LDP at RA and BB [4.0% (0–35.5) and 3.8% (0–28.3)] was considerably higher than at the PVA [1.6% (0–78.1); *P* < 0.001 and *P* = 0.001] and LA [1.7% (0–35.8); *P* < 0.001 for both]. The proportion of FP was also highest at BB compared to all other atrial regions [BB: 1.4% (0–14.8) vs. RA: 1.3% (0–14.8), LA: 1.3% (0–16.2), PVA: 0.7% (0–11.8); *P* < 0.008 for all].

**FIGURE 4:**
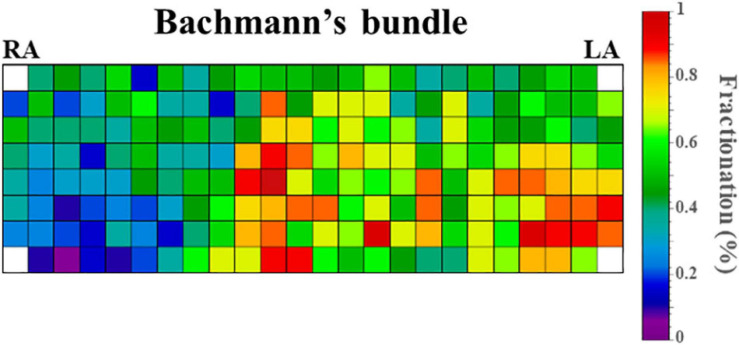
Relative incidence of fractionated potentials at Bachmann’s bundle of a subset of 123 patients who were mapped with the 192-electrode array. LA, left atrium; RA, right atrium.

Figure 4 demonstrates the relative incidence of FPs at BB in a subset of patients [*N* = 123 (65%)] who were mapped with the 192-electrode array which covered the whole BB region. Although FPs could be found over the entire mapping area, most FPs were recorded near the center of BB and its entrance to the LA.

**FIGURE 5:**
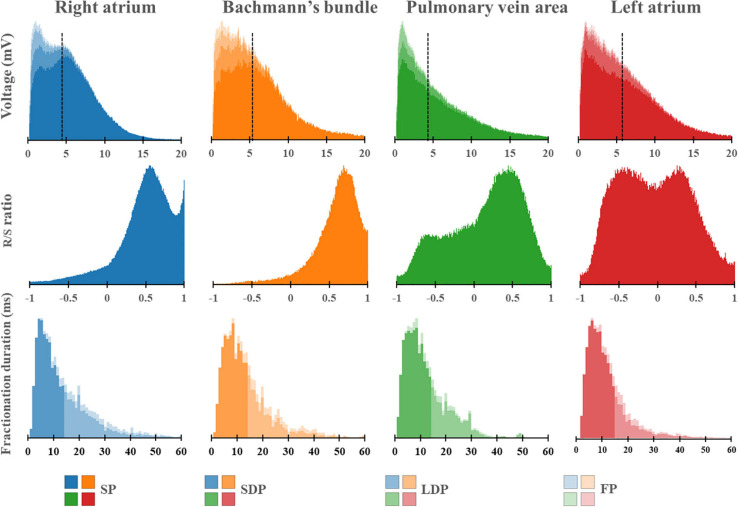
Histograms of the relative frequency distribution of all voltages **(upper)**, R/S ratios **(middle)**, and fractionation durations **(lower)** recorded at the right atrium (blue), Bachmann’s bundle (orange), pulmonary vein area (green), and left atrium (red). Median voltage values are indicated by the black dotted lines. All histograms are subdivided and stacked according to the potential type classification (SP, SDP, LDP, or FP). SP, single potential; SDP, short double potential; LDP, long double potential; FP, fractionated potential.

Figure 5 shows regional differences in the features of the various EGM types. Histograms in the upper panel show the relative frequency distribution of voltages of SP, SDP, LDP, and FP separately obtained from the RA, BB, PVA, and LA. The lowest median voltages were recorded at the PVA [RA: 4.68 mV (IQR: 2.39–7.33); BB: 5.29 mV (IQR: 2.75–8.65); LA: 5.38 mV (IQR: 2.68–9.18); PVA 4.55 mV (IQR: 2.14–8.72)].

The RA region has the highest proportion of low-voltage EGMs [RA: 5.75% (IQR: 2.31–10.64), LA: 4.47% (IQR: 1.26–9.53), PVA: 4.34% (1.23–15.87), and BB: 2.20% (IQR: 0.55–8.43)].

The middle panel of Figure 5 shows histograms of the relative frequency distribution of R/S ratios of SP for the four different atrial regions separately. These R/S ratios differed significantly between the four atrial regions [BB: 0.63 (IQR: 0.55–0.71), RA: 0.54 (IQR: 0.47–0.60), PVA: 0.25 (IQR: 0.02–0.40), and LA −0.02 (IQR: −0.21–0.20); *P* < 0.001 for each]. SPs at the RA and BB had a clear predominant S-wave morphology (ratio R- and S-wave: RA 1:2 and BB 1:2.5). In addition, there was also a large number of SP consisting mainly of solely an S-wave at the RA. In contrast to the RA and BB, SPs recorded from the LA and PVA had a more widespread distribution of R/S ratios. The histogram of the LA had a bimodal distribution with a preference for both predominant R-wave (2:1) and predominant S-wave morphology (1:1.7), whereas the histogram derived from the PVA contained a clear second peak at predominant S-waves (1:2).

Histograms in the lower panel show the relative frequency distribution of FD of the SDP, LDP, and FP separately. FP with the longest FD was recorded at BB [FD BB: 11.0 ms (IQR: 9.0–14.0); RA: 10.0 ms (IQR: 8.0–14.0), PVA: 9.0 ms (IQR: 8.0–12.0), and LA: 9.0 ms (IQR: 7.0–12.0), *P* < 0.001 for each].

### Correlations Between Clinical Characteristics and EGM Features

Supplementary Table 1 shows the correlations between clinical characteristics and EGM features for all regions combined and for each region separately. As can be seen in this table, correlations are either not significant or weak.

### Correlations Between Conduction Block and EGM Features

**FIGURE 6:**
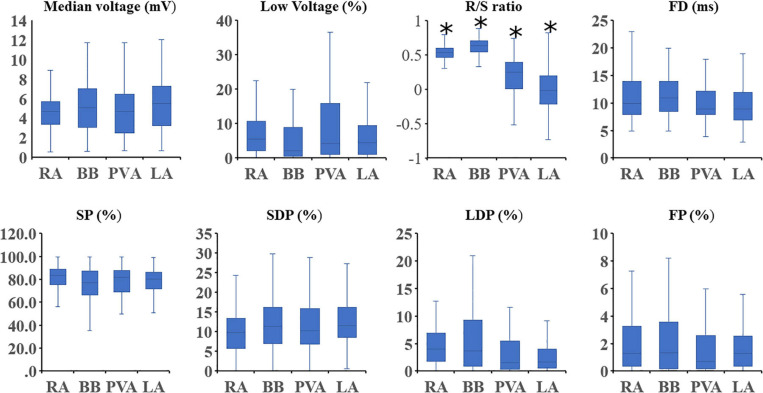
Boxplots depicting the median value of the EGM features for each region separately. Statistical significance for comparison between all other atrial regions is indicated by an asterisk. SP, single potential; SDP, short double potential; LDP, long double potential; FP, fractionated potential; FD, fractionation duration.

Figure 6 shows that for every atrial region, there is considerable interindividual variation of each EGM feature. These EGM features were correlated with the amount of CB. As expected, there was a strong positive correlation between the prevalence of CB and the proportion of LDP (*r* = 0.70) and FP (*r* = 0.53). Table 3 summarizes Spearman’s rank correlation coefficients between CB and EGM features for the four atrial regions separately. CB was strongly correlated with the proportion of LDP at all atrial regions and with the proportion of FP at RA, BB, and PVA. Likewise, FD was correlated with CB at the RA, BB, and PVA. The presence of low-voltage areas was strongly related with the occurrence of CB at RA and BB. The strongest correlation was found between CB and the proportion of LDP at the RA (*r* = 0.83).

**TABLE 3:** Regional correlations between conduction block and EGM features.

**Variables**	**RA**		**BB**		**PVA**		**LA**	
	**Median (min-max)**	**Rho (CB)**	**Median (min-max)**	**Rho (CB)**	**Median (min-max)**	**Rho (CB)**	**Median (min-max)**	**Rho**
CB (%)	2.4 [1.2–3.8]		2.3 [1.0–5.1]		1.1 [0.2–2.8]		0.7 [0.2–1.7]	
Median voltage (mV)	4.68 [3.43–5.72]	−0.47	5.07 [3.16–6.99]	−0.64	4.73 [2.52–6.44]	−0.54	5.54 [3.28–7.30]	−0.05*
Low voltage (%)	5.75 [2.31–10.64]	0.69	2.20 [0.55–8.43]	0.73	4.34 [1.23–15.87]	0.57	4.47 [1.26–9.53]	0.14*
R/S ratio	0.54 [0.47–0.60]	0.20	0.63 [0.55–0.71]	0.16	0.25 [0.02–0.40]	0.08*	−0.02 [−0.21–0.20]	0.22
SP (%)	83.7 [75.7–89.3]	−0.65	77.4 [67.3–87.3]	−0.59	81.9 [69.5–88.1]	−0.51	80.6 [72.0–86.5]	−0.41
SDP (%)	9.8 [5.9–13.4]	0.28	11.5 [7.2–16.3]	0.33	10.3 [6.9–16.0]	0.12*	11.6 [8.7–16.2]	0.18
LDP (%)	4.0 [1.9–6.8]	0.83	3.8 [0.9–9.1]	0.80	1.6 [0.3–5.5]	0.82	1.7 [0.6–4.1]	0.61
FP (%)	1.3 [0.4–3.2]	0.64	1.4 [0.2–3.6]	0.69	0.7 [0.2–2.5]	0.64	1.3 [0.4–2.5]	0.46
FD (ms)	10.0 [8.0–14.0]	0.73	11.0 [9.0–14.0]	0.68	9.0 [8.0–12.0]	0.77	9.0 [7.0–12.0]	0.59

*Values are presented as or median [interquartile ranges]. Rho values with asterisk are not statistically significant.RA, right atrium; BB, Bachmann’s bundle; PVA, pulmonary vein area; LA, left atrium; CB, conduction block; SP, single potential; SDP, short double potential; LDP, long double potential; FP, fractionated potential; FD, fractionation duration.*

**FIGURE 7:**
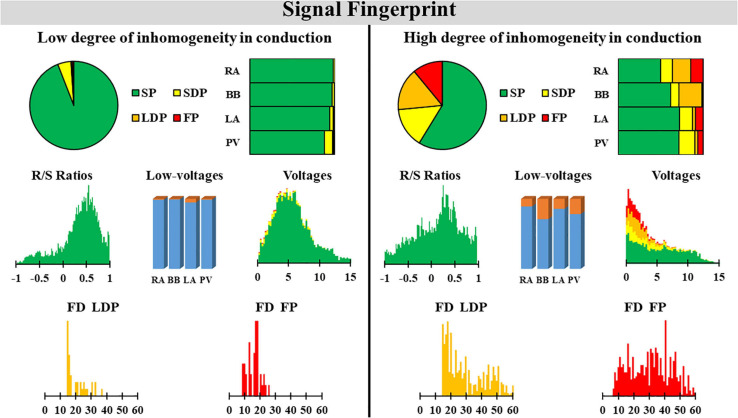
Two examples of a signal fingerprint obtained from one patient with a low and one with a high degree of inhomogeneity in conduction. The upper panels show the distribution of potential types of the entire (**left**, pie plot) and for each region separately (**right**, stacked bars). The middle left plot shows the R/S ratio distribution of all SPs of the entire atrium. The middle center bar plot displays the number of low-voltage potentials plotted for each region separately. The middle right plot shows the distribution of voltages (mV) of SPs (green), SDPs (yellow), LDPs (orange), and FPs (red) from all atrial regions. The lower panel displays the distribution of fractionation duration (ms) for LDPs **(left)** and FPs **(right)**. SP, single potential; SDP, short double potential; LDP, long double potential; FP, fractionated potential; FD, fractionation duration.

In order to test whether various degrees of inhomogeneity in conduction indeed translate into EGM morphology on the individual level, we compared EGM features between two patients with a high and low degree of inhomogeneous conduction. These examples of the resulting electrical signal fingerprints consisting of all quantified EGM features as described above are shown in Figure 7.

## Discussion

### Key Findings

This is the first report on quantification of EGM properties measured during SR at a high-resolution scale throughout the RA and LA including BB. Data were obtained from a large cohort of patients without a history of atrial tachyarrhythmias. In all atrial regions, the majority of the EGMs consisted of SP; the highest proportion of LDP and FP was mainly recorded at the RA and BB; fractionation at BB also had the longest durations. The largest prevalence of low-voltage areas was found at the RA. CB was correlated with the proportion of LDP and FP, FD of FP, and the prevalence of low-voltage areas. Thus, conduction inhomogeneity can be identified by a signal fingerprint containing specific quantified EGM features.

### Relation Between R/S Ratio and Conduction Inhomogeneity

Electrogram morphology of SPs, mainly determined by the magnitude of the R- and S-wave, contains information on conduction inhomogeneity. The unipolar EGM morphology represents the sum of instantaneous current dipoles of a propagating wavefront, generating a positive deflection (R-wave) and negative deflection (S-wave) as the activation wavefront propagates, respectively, toward and away from the recording electrode (Spach et al., 1979; Stevenson and Soejima, 2005). This results in the typical R-S morphology of the majority of the SPs. Certain EGM morphologies are found at specific atrial regions. For example, wavefront propagation during normal SR is initiated at the sino-atrial node from where it spreads in contiguous, prominent muscle bundles. When recording EGMs near this area, the activation wavefront only propagates away from the recording electrodes resulting in SPs consisting of solely S-wave morphologies. As intra-atrial conduction during SR is generally characterized by smooth and fast propagation, the majority of the SPs, however, have a typical large amplitude R-S morphology.

van der Does et al. (2018) performed high-resolution mapping of the RA wall and evaluated differences in EGM morphology of >2,000 pairs of simultaneously acquired endo- and epicardial EGMs. There was no difference in R/S ratios of opposite endo- and epicardial EGMs, and both sites showed a predominant S-wave morphology. The cause of S-wave predominance was further investigated by van Schie et al. (2020). They investigated the impact of AF episodes on SP morphology. In patients with AF, there was a loss of S-wave amplitude resulting in SPs with lower amplitudes and shifted R/S ratios. This reduction of S-wave amplitude was also associated with a decrease in conduction velocity. Hence, these observations indicate that the R/S ratio of SPs is a suitable marker of conduction inhomogeneity and it should therefore be incorporated in the diagnostic electrical signal fingerprint.

### Relation Between Fractionation, Fractionation Duration, and Conduction Inhomogeneity

Fractionation of EGMs is caused by asynchronous activation of adjacent cardiomyocytes, due to the presence of structural barriers such as fibrotic strands. This in turn gives rise to inhomogeneous intra-atrial conduction. In addition, increased slowing of intra-atrial conduction is related to prolonged duration of FPs (de Bakker and Wittkampf, 2010; van der Does and de Groot, 2017).

Konings et al. (1997) were the first to demonstrate the relation between EGM morphology and specific patterns of activation. During induced AF in patients with the Wolf-Parkinson White syndrome, SDPs were mainly caused by collision of fibrillation waves, LDPs were recorded along long lines of CB and FP in areas of slow conduction or at pivot points. Collision of wavefronts may also occur during SR, for example, when the SR wavefront divides to turn around small barriers such as tissue discontinuities caused by fibrotic strands and merges again at the opposite sites.

The relation between LDP and FP and patterns of activation has also been investigated during SR. Simultaneous endo-epicardial high-resolution mapping studies demonstrated that fractionation of unipolar EGMs during SR is not only the result of slowing of conduction or pivoting of the wavefront around a line of CB, but that it can also be attributed to asynchronous activation of the endo- and epicardial wall. Even during SR, asynchronous activation up to 84 ms has been described (Kharbanda et al., 2020).

Inhomogeneous conduction and hence EGM fractionation may also be caused by normal properties of the myocardial tissue. For example, the trabeculated parts of the atria contain muscle bundles of variable thickness. As a consequence, small myocardial strands excite larger myocardial areas leading to source-to-sink mismatches which also cause slowing of conduction. This explains recordings of LDP and FP during SR in all patients. In a series of prior mapping studies of the atria during SR, it was demonstrated that lines of CB most frequently occur at the superior part of the RA (sino-atrial node area) and BB, which is consistent with the high proportion of LDP and FP observed in the present study (Teuwen et al., 2016; Kharbanda et al., 2020).

### Relation Between Unipolar Voltages and Conduction Inhomogeneity

Peak-to-peak amplitudes of unipolar EGM are affected by numerous variables including not only tissue-related factors, but also to EGM recording and processing technologies. In general, areas of low voltages are considered as surrogate markers of fibrotic tissue and have therefore become targets for ablative therapy in patients with AF (Rolf et al., 2014; Kottkamp et al., 2015; Yagishita et al., 2017). This even includes low-voltage areas identified during SR. Regional differences in unipolar voltages have recently been investigated in a cohort of 67 patients with mitral valve disease undergoing cardiac surgery by van Schie et al. (2021). Not only marked inter-individual differences in unipolar voltages, but also regional differences in unipolar voltages were observed. Patients with AF had lower EGM voltages during SR, particularly at BB. There were also regional differences in the proportion of low-voltage EGMs; the lowest and highest number of low-voltage EGMs were found at, respectively, BB (2.20%) and RA (5.75%). Comparable to the patients with mitral valve disease, we also observed in our patients with coronary artery disease the highest proportion of low-voltage EGMs at the RA and BB. The wide distribution of low-voltage areas particularly at RA, BB, LA, and PVA may explain why ablation approaches in patients with AF targeting low-voltages areas during SR solely in the LA may not be beneficial.

### Mapping of Right Atrium

Current ablation procedures mostly consist of a pulmonary vein isolation and focus on the LA. However, other procedures like the Cox-Maze include the RA as well. Prior studies have demonstrated that the RA is driving AF in ≈20% of persistent AF (Hocini et al., 2010). Thus, the signal fingerprints of the RA should not be ignored. We performed mapping of the cavotricuspid isthmus area. Thus far, we have no additional evidence that the cavotricuspid isthmus is a major player in the pathophysiology of AF or that it is a predilection site for electropathology in these patients. As described above, the highest proportion of LDP and FP was mainly recorded at the RA and BB, and the largest prevalence of low-voltage areas was found at the RA, which might partly explain why catheter ablation based on low-voltage areas or FP at RA region cannot achieve ideal benefits. Since low-voltage potentials and fractionation have been recorded during SR, it is still challenging to identify whether this phenomenon is caused by physiology or pathology. This should be a new direction for subsequent studies.

### Limitations

The high-resolution epicardial mapping is an invasive approach and cannot easily be translated to non-invasive data. The impact of endo-epicardial asynchrony on differences between the endo- and epicardial fingerprint is at present unknown and needs to be further investigated.

## Conclusion

At present, we have no diagnostic tool to determine the severity and extensiveness of conduction inhomogeneity, which plays a major role in initiation and perpetuation of atrial tachyarrhythmias, including AF. The signal fingerprint, consisting of quantified EGM features, including the R/S ratio of SPs, the relative frequency distribution of unipolar voltages, the proportion of low-voltage areas, the proportion of the different types of EGMs, and durations of LDP and FDP, serves as a marker of the severity and extensiveness of conduction inhomogeneity. Additional studies are required to further develop the signal fingerprint in order to identify patients at risk for AF onset or progression. The invasively determined signal fingerprint will serve as a golden standard in less- or even non-invasive fingerprints.

## Data Availability Statement

The datasets presented in this article are not readily available because of EU privacy law. Requests to access the datasets should be directed to NG, n.m.s.degroot@erasmusmc.nl.

## Ethics Statement

The studies involving human participants were reviewed and approved by MEC2010-054/MEC2014-393. The patients/participants provided their written informed consent to participate in this study.

## Author Contributions

ZY and MS contributed to data analysis, manuscript drafting, and conceptual thinking. NG contributed to manuscript drafting and conceptual thinking. All authors contributed to the article and approved the submitted version.

## Conflict of Interest

The authors declare that the research was conducted in the absence of any commercial or financial relationships that could be construed as a potential conflict of interest.
