# Supplementary figures and images for: Signal Fingerprinting as a Novel Diagnostic Tool to Identify Conduction Inhomogeneity
Source: Front Physiol. 2021 Mar 26;12:652128. doi: 10.3389/fphys.2021.652128 (PMC8033016; doi:10.3389/fphys.2021.652128)

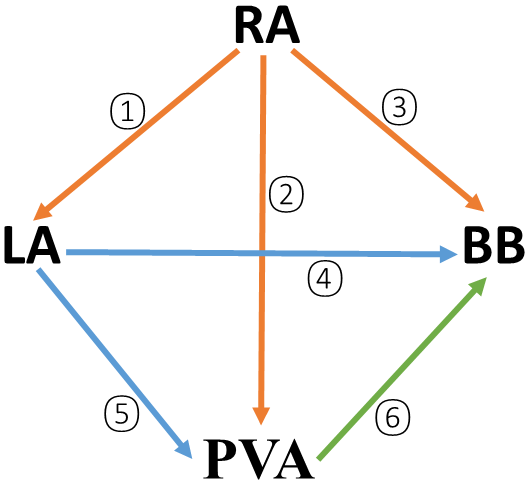

Supplement: Supplementary file 1 [file Image_1.TIF]
