# Supplementary material for: Signal Fingerprinting as a Novel Diagnostic Tool to Identify Conduction Inhomogeneity
Source: Front Physiol. 2021 Mar 26;12:652128. doi: 10.3389/fphys.2021.652128 (PMC8033016; doi:10.3389/fphys.2021.652128)
Supplement: Supplementary file 2 [file Table_1.DOCX]

**Supplementary Table S1. Correlation analysis of signal features and baseline characteristics**

| Variables | Median voltage | Low-voltage | Mean R/S ratio | SP | SDP | LDP | FP | FD |
| --- | --- | --- | --- | --- | --- | --- | --- | --- |
| **All regions** |  |  |  |  |  |  |  |  |
| Age | **-0.264*** | **0.242*** | NS | NS | NS | NS | NS | NS |
| Sex | NS | NS | NS | NS | NS | NS | NS | NS |
| BMI | NS | NS | NS | NS | NS | NS | NS | NS |
| Hypertension | NS | NS | NS | NS | NS | NS | NS | NS |
| Dyslipidemia | NS | NS | NS | NS | NS | NS | NS | NS |
| Diabetes mellitus | NS | NS | NS | NS | NS | NS | NS | NS |
| Left ventricular dysfunction | NS | NS | NS | NS | NS | NS | NS | NS |
| Left atrial dilatation >45 mm | NS | NS | NS | NS | NS | NS | NS | NS |
| **RA** |  |  |  |  |  |  |  |  |
| Age | **-0.237*** | **0.247*** | **-0.183*** | NS | NS | NS | NS | NS |
| Sex | NS | NS | NS | NS | NS | NS | NS | NS |
| BMI | NS | NS | **0.175*** | NS | NS | NS | NS | NS |
| Hypertension | NS | NS | NS | NS | NS | NS | NS | NS |
| Dyslipidemia | NS | NS | NS | NS | NS | NS | NS | NS |
| Diabetes mellitus | NS | NS | NS | NS | NS | NS | NS | NS |
| Left ventricular dysfunction | NS | NS | NS | NS | NS | NS | NS | NS |
| Left atrial dilatation >45 mm | NS | NS | NS | NS | NS | NS | NS | NS |
| **BB** |  |  |  |  |  |  |  |  |
| Age | **-0.277*** | **0.228*** | NS | NS | NS | **0.174*** | NS | NS |
| Sex | NS | NS | NS | NS | NS | NS | NS | NS |
| BMI | NS | NS | NS | NS | NS | NS | NS | NS |
| Hypertension | NS | NS | NS | NS | NS | NS | NS | NS |
| Dyslipidemia | NS | NS | NS | NS | NS | NS | NS | NS |
| Diabetes mellitus | NS | NS | NS | NS | NS | NS | NS | NS |
| Left ventricular dysfunction | NS | NS | NS | NS | NS | NS | NS | NS |
| Left atrial dilatation >45 mm | NS | NS | NS | NS | NS | NS | NS | NS |
| **PVA** |  |  |  |  |  |  |  |  |
| Age | NS | NS | NS | NS | NS | NS | NS | NS |
| Sex | NS | NS | NS | NS | NS | NS | NS | NS |
| BMI | NS | NS | NS | NS | NS | NS | NS | NS |
| Hypertension | NS | NS | NS | NS | NS | NS | NS | NS |
| Dyslipidemia | NS | NS | NS | NS | NS | NS | NS | NS |
| Diabetes mellitus | NS | NS | NS | NS | NS | NS | NS | NS |
| Left ventricular dysfunction | NS | NS | NS | NS | NS | NS | NS | NS |
| Left atrial dilatation >45 mm | NS | NS | NS | NS | NS | NS | NS | NS |
| **LA** |  |  |  |  |  |  |  |  |
| Age | NS | NS | NS | NS | NS | NS | NS | NS |
| Sex | NS | NS | NS | NS | NS | NS | NS | NS |
| BMI | NS | NS | **-0.168*** | NS | NS | NS | NS | NS |
| Hypertension | NS | NS | NS | NS | NS | NS | NS | NS |
| Dyslipidemia | NS | NS | NS | NS | NS | NS | NS | NS |
| Diabetes mellitus | NS | NS | NS | NS | NS | NS | NS | NS |
| Left ventricular dysfunction | NS | NS | NS | NS | NS | NS | NS | NS |
| Left atrial dilatation >45 mm | NS | NS | NS | NS | NS | NS | NS | NS |

RA=right atrium; BB= Bachmann’s bundle; PVA=pulmonary vein area; LA=left atrium; BMI = body mass index; SP = single potential; SDP = short double potential; LDP = long double potential; FP = fractionated potential; FD = fractionation duration. NS=no significance.

*indicate p value <0.05.
